# Prostaglandin E2 receptor EP1 expression in vulvar cancer

**DOI:** 10.1007/s00432-022-04487-z

**Published:** 2022-11-27

**Authors:** Anna Buchholz, Aurelia Vattai, Sophie Fürst, Theresa Vilsmaier, Alaleh Zati Zehni, Alexander Steger, Christina Kuhn, Elisa Schmoeckel, Christian Dannecker, Sven Mahner, Udo Jeschke, Helene H. Heidegger

**Affiliations:** 1 Department of Obstetrics and Gynecology, University Hospital, LMU Munich, Marchioninistrasse 15, 81377 Munich, Germany; 2 Klinik und Poliklinik für Innere Medizin I, University Hospital, Technical University of Munich, Ismaninger Straße 22, 81675 Munich, Germany; 3 Department of Obstetrics and Gynecology, University Hospital Augsburg, Augsburg, Germany; 4 Department of Pathology, LMU Munich, Thalkirchner Str. 142, 80337 Munich, Germany

**Keywords:** EP1, PGE2, COX-2, Vulvar carcinoma, Cancer survival, Prognosis

## Abstract

**Purpose:**

In recent years, incidence of vulvar cancer has been on the rise, whereas therapeutic options are still restricted. Therefore, new prognosticators and therapeutic targets are essential. Chronic inflammation plays an important role in carcinogenesis and COX-2, and its product prostaglandin E2 and its receptors EP1–4 are known to be important mediators in cancer initiation and progression.

**Methods:**

EP1 expression in vulvar cancer specimens (*n* = 129) was investigated via immunohistochemistry and evaluated using the well-established immunoreactive score (IRS). Subsequently, the values were correlated with clinicopathological parameters.

**Results:**

Our analysis did not reveal EP1 expression as a negative prognostic factor in overall and disease-free survival. However, in the subgroup of patients with lymph-node metastasis, overall survival was significantly shorter in tumors with high EP1 expression. Moreover, EP1 expression correlated positively with good differentiation of the tumor, but not with p16 status or COX-2 expression.

**Conclusions:**

This study shed first light on EP1 expression in vulvar carcinoma. EP1 expression correlated significantly with the grading of the tumor, suggesting that it influences cell differentiation. Further research on EP1 signaling may lead to a deeper understanding of the molecular mechanisms of carcinogenesis.

## Introduction

Vulvar carcinoma is a relatively rare disease, being the fifth most common gynecological tumor worldwide (International Agency for Research on Cancer 2020). Squamous cell carcinoma (SCC) is its most common histologic subgroup, representing around 90% of all tumors of the vulva (Kang et al. 2017). Incidence rates have been on the rise in recent years, possibly caused partly due to an increasing prevalence of human papilloma virus (HPV) infection likely promoted by a social change in sexual behavior (Bray et al. 2020). The HPV-related pathway leading to the development of around 40% of all vulvar squamous cell carcinoma (VSCC) can be distinguished from an HPV independent pathway often associated with chronic inflammatory dermatological diseases such as lichen sclerosis or lichen planus (Carlson et al. 1998; Regauer et al. 2014; Smith et al. 2009). Radical local surgical resection often accompanied by lymphonodectomy and adjuvant radiotherapy is the predominantly applied therapy for both tumor entities, while systemic therapies are only marginally administered resulting from a lack of randomized controlled trials (Mahner et al. 2015). Despite limited literature regarding long-term effects of vulvar cancer therapy, radical surgery often involving large resection areas up to vulvectomy can lead to reduced quality of life with impaired sexual function, lymph edema, or urinary difficulties (Gitas et al. 2021; Pilger et al. 2012; Ramaseshan et al. 2018). Although HPV vaccination gives the opportunity for primary prevention of a smaller proportion of VSCC, further risk factors such as smoking habits and an aging population additionally support the need for new predictive biomarkers and target-based therapies to ameliorate the clinical outcome for patients, especially in late stages of the disease (Daling et al. 1992; Joura et al. 2007). Currently, possible therapy targets are vascular endothelial growth factor (VEGF) and epidermal growth factor receptor (EGFR) (Mahner et al. 2015). VEGF is an important mediator for angiogenesis and when overexpressed has a negative impact on the prognosis of vulvar carcinoma patients (Obermair et al. 1996). EGFR is a transmembrane protein and belongs to the family of receptor tyrosine kinases. It is often overexpressed in a vulvar carcinoma, and is associated with decreased survival, high tumor stage, lymph-node metastasis, and HPV-negativity (Growdon et al. 2008; Woelber et al. 2012). There are few case reports describing clinical benefits from the treatment with monoclonal antibody Cetuximab (Bergstrom et al. 2015; Matsuzawa et al. 2016). Also, EGFR inhibitor Erlotinib showed overall clinical benefit in 67.5% of patients in a phase II clinical trial with 41 patients. Nevertheless, therapy with the EGFR inhibitor implicated significant grade 3/4 toxicity including allergic reaction, diarrhea with electrolyte abnormalities, ischemic colitis, and renal failure, and often is often accompanied by early resistance to therapy (Horowitz et al. 2012). Therefore, the search for new prognostic markers and possible targets is necessary.

As chronic inflammation is known to play an important role in cancer initiation, progression, angiogenesis, and metastasis, prostanoid metabolism presents an interesting research topic in different tumor entities (Shacter and Weitzman 2002). Previous studies investigated the function of cyclooxygenase enzyme 2 (COX-2), prostaglandin E2 (PGE2), and its receptors (EP) in many different tumor entities like colon cancer, prostate and lung cancers, as well as in breast cancer and gynecological tumors, such as ovarian, cervical, and endometrial cancers (Howe 2007; Wang and Dubois 2010a, 2010b; Ye et al. 2020). PGE2 is produced from arachidonic acid by cyclooxygenase enzyme 2 (COX-2) and mediates its effects through its specific G-protein coupled receptors EP1–EP4, whereas EP1 is studied the least (Ye et al. 2020). Affinity for PGE2 binding is substantially different between the EP receptor subtypes with the following rank order of affinities: EP3 > EP4 >  > EP2 > EP1 (Abramovitz et al. 2000). Each receptor induces different signaling cascades, e.g., through the intracellular increase of Ca^2+^ and subsequently activation of protein kinase C (EP1) or the increase of secondary messenger cAMP levels and activation of protein kinase A (EP2 and EP4) (O’Callaghan and Houston 2015). EP1 is involved in several different signaling pathways. Previous research indicates the involvement of EP1 in an EGFR independent activation of the MAPK/ERK pathway in non-small cell cancer of the lung (Krysan et. al. 2005). Pan et al. demonstrated the regulatory role of EP1 on β1-integrin through the COX-2/EP1/MAPK/E2F-1 pathway (Pan et al. 2016). Moreover, EP1 seems to mediate enhanced matrix metalloproteinase-2 (MMP-2) expression by cAMP independent CREB phosphorylation, illustrating its importance in cancer invasion (Sun et. al. 2013). A study on hepatocellular carcinoma cells shows that EP1 promotes cell adhesion and migration by inducing phosphorylation of focal adhesion kinase (FAK) (Bai et al. 2013). Furthermore, in a in vivo study using a prostate cancer mouse model, mice that were treated with an EP1 antagonist showed a significantly lower incidence of cancer at simultaneously a higher percentage of apoptotic cells compared to control (Masato et. al. 2021).

The expression and function of COX-2, PGE 2, and EPs in vulvar cancer have been subject to little research to date. Results from previous studies identified combined cytoplasmatic COX-2 expression and EP4 expression as negative prognostic factors for survival in vulvar cancer patients. Furthermore, positive EP4 expression correlated significantly with higher FIGO stage and tumor size (Ansorge et al. 2021; Buchholz et al. 2021). The aim of this study is to examine the expression of EP1 in vulvar cancer via immunohistochemistry and to analyze its correlation with clinicopathologic variables and its effect on patients’ survival to possibly find new prognostic markers and possible therapeutic targets.

## Methods

### Patients collective

The patients’ collective was composed of 177 patients who were treated at the Department of Gynecology and Obstetrics of the Ludwig-Maximilians-University in Munich, Germany between 1990 and 2008. During surgery, collected tissue material was histopathological processed and specified. Munich Cancer Registry (MCR) from the Munich Tumor Centre (TZM—Munich Tumor Centre, Munich, Germany) provided the follow-up and survival data. For immunohistochemical staining, 157 of these 177 tissue samples were accessible. During microscopic analysis, another 28 samples were excluded, as the specific tissue slices did not contain cancer tissue. In the end, 129 patients were included in the statistical analysis. Patients’ median age was 69.5 years (range 20–96 years) and overall median survival was 7.03 years. Other relevant patient characteristics are displayed in Table 1.

**Table 1:** Clinicopathological characteristics of the patients collective

Clinicopathologic parameters		*n*	Percentage (%)
Histology	Keratinizing	160	90.4
	Warty/basaloid	17	9.6
Tumor size	T1	69	39
	T2	92	52
	T3	9	5.1
	Missing	7	3.9
Nodal status	N0	78	44.1
	N	38	21.5
	N	12	6.8
	Missing	49	27.6
Metastasis	M0	8	4.5
	Missing	169	95.5
Figo	I	61	34.4
	II	54	30.5
	III	47	26.6
	IV	9	5.1
	Missing	6	3.4
Grading	G1	29	16.4
	G2	108	61
	G3	39	22
	Missing	1	0.6
p16 status	Positive	38	21.5
	Negative	57	32.3
	Missing	82	46.3

### Immunohistochemistry

Formalin fixated and paraffin-embedded samples were cut to 4 µm slices, before being mounted on SuperFrost Plus microscope slides (Menzel Glaeser, Braunschweig, Germany). Staining was conducted as previously similarly described (Buchholz et al. 2021). First, sample slides were deparaffined in xylol for 20 min and washed in 100% alcohol. To stop the activity of endogen peroxidases, samples were incubated in methanol with 3% H_2_O_2_ for 20 min. Afterward, the slides were rehydrated in descending alcohol (100%, 70%, and 50%) and washed with distilled water. In the use of a pressure cooker, samples were heated in sodium citrate buffer (pH = 6.0) (0.1 M citric acid and 0.1 M sodium citrate in distilled water) to unmask antigens, which agglomerate during formalin fixation. After cooling and washing in PBS, slides were prepared with a blocking solution (Power Block, Biogenex Laboratories, Fremont, CA, USA) for 5 min to saturate electrostatic charges and avoid nonspecific hydrophobic binding of the primary antibodies. In the next step, the primary anti-EP1 antibody dilution (1:300 in PBS) (polyclonal rabbit IgG; AB217925, Abcam, Cambridge, UK) was applied and the slides were incubated for 16 h at 4 °C in a humidity chamber. ZytoChem Plus HRP Polymer System (Zytomed, Berlin, Germany) was used for detection via secondary complex. It contained the application of a post-block reagent for 20 min and an HRP-polymer for 30 min thereafter, both at room temperature in the humidity chamber. In the end, coloration of the substrate was catalyzed by the chromogen diaminobenzidine (Dako, Hamburg, Germany) and stopped with H_2_O. In a last step, slides were counterstained with hemalaun for 2 min and washed in ascending alcohol and covered with glass. EP1 expression was evaluated with a Leitz microscope (Wetzlar, Germany) using the well-established semiquantitative immunoreactivity score (IRS). Therefore, a product of two factors is formed: the intensity of the staining (0 = no, 1 = weak, 2 = moderate, and 3 = strong staining) multiplied by the percentage of stained cells (0 = no staining, 1 = 10% positive cells, 2 = 11–50% positive cells, and 3 = 50% positive cells) (Remmele and Stegner 1987). Ultimately, the IRS score of the samples was correlated with clinicopathological parameters and groups with high and low EP1 expression were compared for progression-free and overall survival. Placenta tissue from the LMU Department of Gynecology and Obstetrics in Munich was utilized as negative and positive control.

### Statistical analysis

Data analysis was performed with the Statistical Product and Service Solutions 28 (PASW Statistic, SPSS Inc., IBM, Chicago, IL, USA). Spearmen’s test was used to test for correlations between immunohistochemically staining and clinicopathological parameters. Nonparametric tests (Mann–Whitney *U*) were used for group comparisons regarding the IRS of the prostaglandin receptors between independent clinical and pathological subgroups and are displayed as boxplot graphs. Survival times were analyzed by Kaplan–Meier curves and log-rank testing (Mantel–Cox). Cut-off points were acquired by the receiver operator curve (ROC). We considered p values ≤ 0.05 as statistically significant.

## Results

**Fig. 1:**
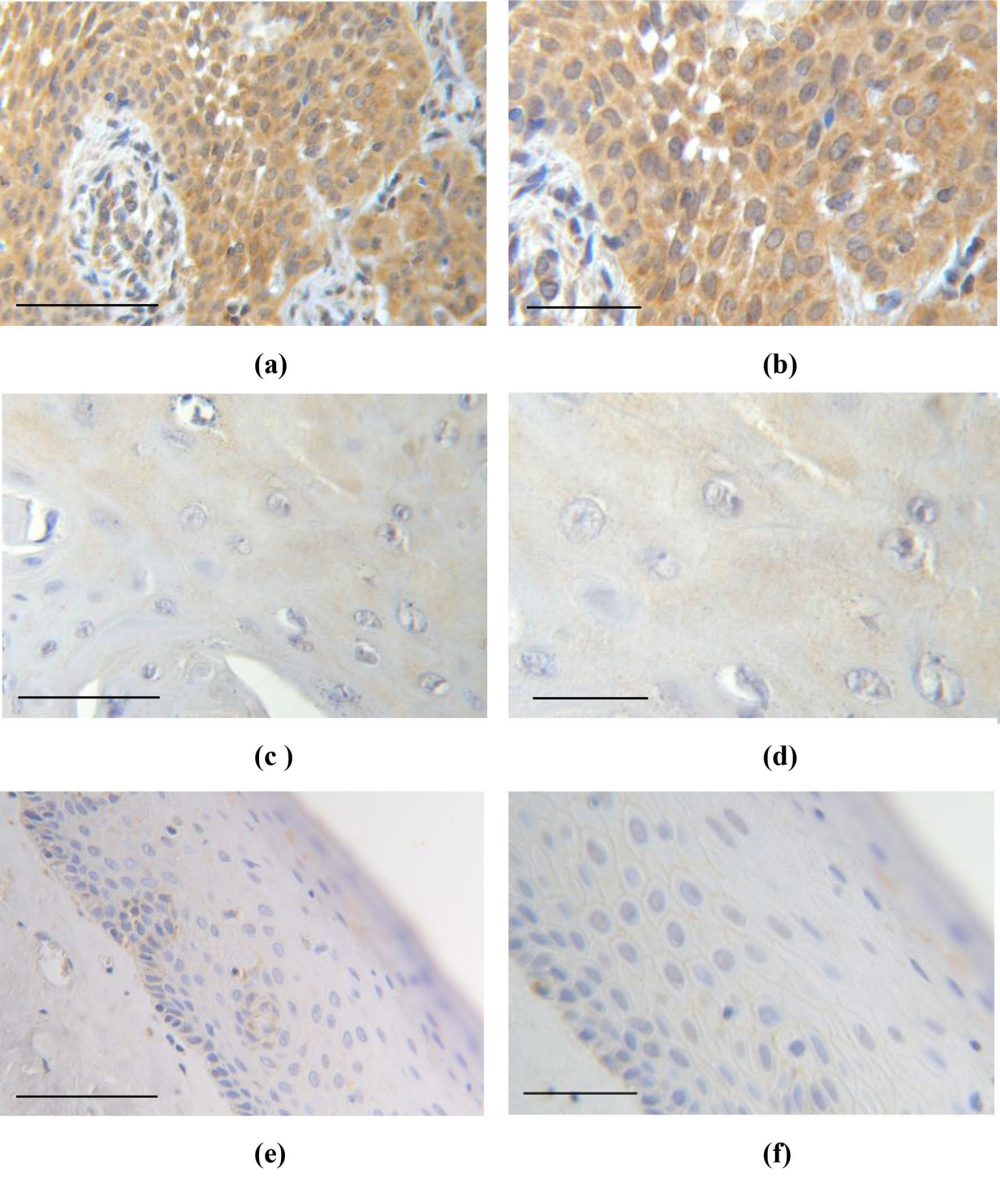
Illustration of different EP1 expression: **a**, **b** Vulvar cancer with high EP1 expression (IRS = 12). **c**, **d** Vulvar cancer with low EP1 expression (IRS = 1–2). **e**, **f** Adjacent benign vulva tissue shows no EP1 expression. Magnification and scale bars (**a**, **c**, **e**) × 25 with scale bar representing 100 µm and (**b**, **d**, **f**) × 40 with scale bar representing 50 µm

We achieved effectual EP1 staining from 129 patients. Cytosolic EP1 (IRS ≥ 1) staining was found in 86.0% (111/129 cases) of cases. High expression (IRS 9–12) was found in 6.2% of specimens, compared to moderate expression (IRS 6–8) in 22.5%, weak expression (IRS 3–4) in 29.5%, and no expression (IRS 0–2) in 41.9% (Fig. 1). In contrast to vulvar cancer specimens, no EP1 expression was found in the adjacent benign tissue of the vulva.

### Correlation of enhanced EP1 expression with shorter overall survival in lymph-node positive patients

**Fig. 2:**
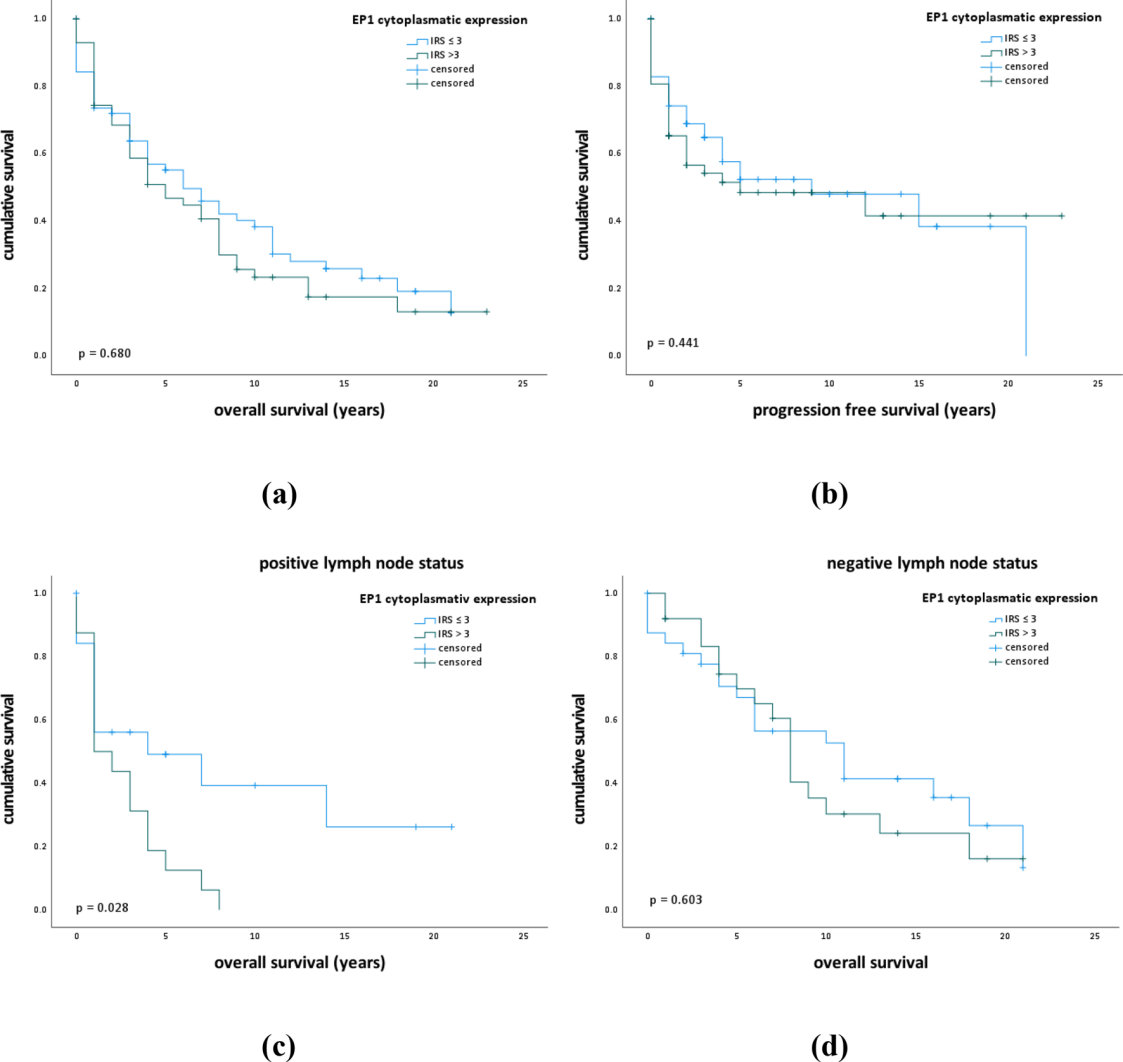
Kaplan–Meier curves display overall and disease-free survival in patients with high and low cytoplasmatic EP1 expression. **a**, **b** Kaplan–Meier survival univariate analysis for the status IRS > 3 showed no significantly diverse overall (*p* = 0.680) or disease-free survival (*p* = 0.441) **c** In the subgroup of patients with lymph-node metastasis, a high EP1 expression (IRS > 3) survival univariate analysis showed significantly shorter overall survival (*p* = 0.028). **d** In the subgroup of patients without lymph-node metastasis, survival univariate analysis showed no significantly diverse overall survival regarding EP1 expression (*p* = 0.603)

Regarding all samples, we could not detect any association between EP1 expression and overall survival (*p* = 0.441) or progression-free survival (*p* = 0.680). However, in the subgroup of patients with lymph-node metastasis, patients with enhanced EP1 expression (IRS > 3) showed significantly worse overall survival than patients with low EP1 expression (IRS ≤ 3) (median estimate: 2.6 years vs. 8.6 years; *p* = 0.028) (Fig. 2).

**Fig. 3:**
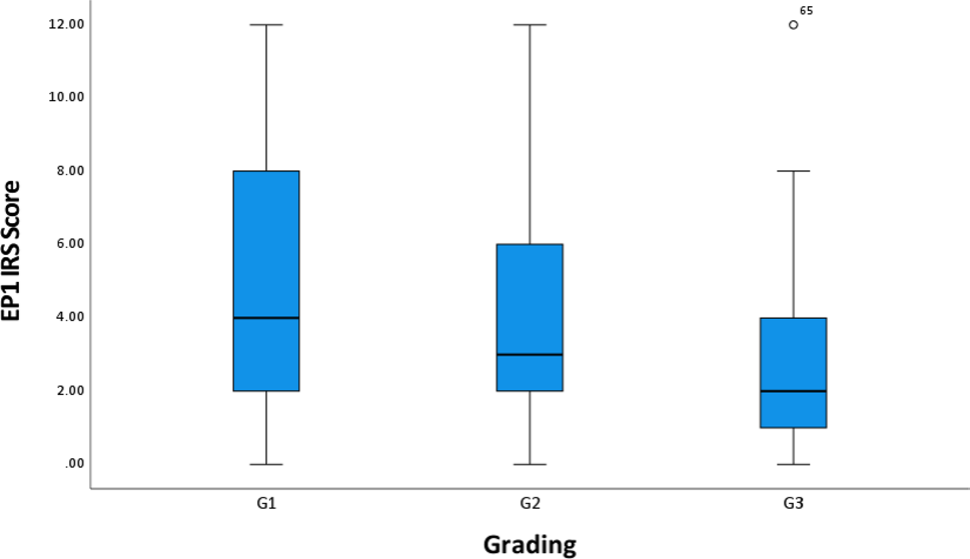
EP1 expression correlates with grading (*p* = 0.031)

In this study, multivariate analysis identified age at diagnosis (*p* = 0.006) and grading (*p* = 0.022) as independent prognostic factors for overall survival. Enhanced EP1 expression, nodal status, tumor size, FIGO classification, or p16 status did not prove to be independent prognosticators (Table 2).

**Table 2:** Cox regression of clinicopathological variables regarding overall survival

	Significance	Hazard ratio of Exp (B)	Lower 95% CI of Exp (B)	Upper 95% CI of Exp (B)
EP1 IRS > 3	0.293	1.337	0.778	2.300
Age	< 0.001	1.055	1.027	1.083
Grading	**0.031**	1.644	1.045	2.586
pN	0.098	1.674	0.909	3.085
pT	0.245	1.825	0.662	5.036
FIGO	0.606	0.817	0.379	1.762
Focality	0.656	0.829	0.363	1.893
Histology	0.713	1.237	0.398	3.844

Bold values characterizes p-values below 0.05

### Correlation of EP1-positive staining with clinicopathologic parameters

Spearmen’s test was used to inspect the correlation between EP1 expression and clinicopathological parameters. We could not detect correlations between EP1 and lymph-node status (*p* = 0.633), tumor size (*p* = 0.807), FIGO classification (*p* = 0.582), the histologic subtype (*p* = 0.523), p16 status (*p* = 0.463), or cytosolic COX-2 expression (*p* = 0.230).

In contrast, EP1 positivity correlated significantly with lower grading of the tumor (*p* = 0.031) (Fig. 3). Mann–Whitney *U* Test confirmed those significant differences in EP1 expression between low-grade (G1) (high expression) and high-grade (G3) (low expression) tumors (*p* = 0.047).

## Discussion

This study shed first light on the expression of G-coupled prostaglandin receptor EP1 in vulvar carcinoma and analyzed its correlation with clinicopathologic parameters and survival. To date, EP1 expression has been described in numerous tumor entities, including breast cancer, endometrial cancer, colon cancer, and skin cancer (Gustafsson et al. 2007; Lee et al. 2005; Thorat et al. 2008; Zhu et al. 2018). Moreover, prostaglandin receptor EP1 is known to be involved in tumor initiation and progression, cell migration and invasion, and the adaption of cancer cells to hypoxia (O’Callaghan and Houston 2015). In comparison to the other EP receptors, the knowledge on EP1 in cancer metabolism is still limited and discussed controversially.

Our results revealed no influence of EP1 on overall or disease-free survival in vulvar cancer patients. These findings are analogous to a study by Zhu et al. They analyzed EP1 in a large collective of endometrial cancer (*n* = 140) and could also not detect any correlation with survival or clinicopathological parameters in endometrial cancer (Zhu et al. 2018). Nonetheless, in our subgroup of patients with lymph-node metastasis, patients with elevated EP1 expression in the tumor had a significantly shorter overall survival.

Since EP1 is known to be involved in cell migration and invasion, it could be possible that EP1 becomes upregulated in tumors during the invasive process of metastasis. Unfortunately, we could not be present during the tissue collecting process; consequently, it was not possible to save lymph-node tissue for the evaluation of EP1 in metastatic tissue.

In addition, we found a correlation of EP1 with tumor grading: well-differentiated tumors showed a significantly higher EP1 expression than poorly differentiated tumors. This result is supported by previous research of EP1 expression in SCC of the skin. Lee et al. found higher EP1 expression, especially in the supra-basal layers of the epidermis in SCC of the skin compared to healthy epidermal tissue (Lee et. al. 2005). EP1 seems to be an important mediator for the regulation of keratinocyte differentiation. This idea is supported by a study of Konger et al. in which they described an inhibition of keratinocyte differentiation after EP1-receptor antagonism in vitro and an enhanced expression of EP1 in well-differentiated SCC compared to poorly differentiated SCC. This regulatory effect seemed to be intact in squamous cell carcinoma of the skin, which is in line with the results from our study (Konger et al. 2009). Furthermore, statistical analysis did surprisingly not reveal any correlation between COX-2 expression [archive data from a previous study by Ansorge et al. (2021)] and EP1 expression. The affinity of EP1 for PGE2 is relatively low; therefore, one could expect an upregulation of COX-2 in EP1-positive tumor samples (Abramovitz et al. 2000). However, previous research showed a posttranslational negative feedback mechanism of EP1 on COX-2 by proteasomal degradation and showed on the other side increased levels of EP1 at COX-2 overexpression, both through transient complex formation (Haddad et al. 2012; Sood et al. 2014).

Finally, some limitations of this study are worth to be noted: on account of the retrospective approach of our study, only one semiquantitative method (immunohistochemistry) was used to evaluate EP1 expression in the patients collective. As we could not be present during the tissue collecting process, it was not possible to save lymph-node tissue for evaluation of EP1 expression and fresh tissue to apply further methods for the analysis of protein activity. Nevertheless, a strength of this study is the large size of the patient collective, considering the rarity of the disease. This study gave first insight in the expression of EP1 in vulvar cancer and its association with clinical data.

## Data Availability

The data presented in this study are available on request from the corresponding author. The data are not publicly available due to ethical issues.

## Abbreviations

cAMP: Cyclic adenosine monophosphate
COX-2: Cyclooxygenase-2
CREB: CAMP response element-binding protein
EGFR: Epidermal growth factor receptor
EP1: Prostaglandin E2 receptor 1
ERK: Extracellular-signal regulated kinase
FAK: Focal adhesion kinase
FIGO: Fédération Internationale de Gynécologie et d’Obstétrique
HPV: Human papilloma virus
IRS: Immunoreactive score
MAPK: Mitogen-activated protein kinases
MMP-2: Matrix metalloproteinase-2
PGE2: Prostaglandin E2
ROC: Receiver-operating characteristic
SCC: Squamous cell carcinoma
VEGF: Vascular endothelial growth factor
VSCC: Vulvar squamous cell carcinoma
